# Morphological and Physiological Changes and Ethylene-Related Gene Expression During Petal Senescence in Red-Flowered Strawberry ‘140’

**DOI:** 10.3390/plants15152412

**Published:** 2026-08-06

**Authors:** Lixiang Miao, Jiyao Qiu, Yijia Ma, Chaocui Nong, Ziping Fan, Rongping Ren, Ming Jiang, Qingxi Chen, Yuji Huang

**Affiliations:** 1 College of Horticulture, Fujian Agriculture and Forestry University, Fuzhou 350002, China; jiyaoqiu2003@163.com (J.Q.); fanziping@163.com (Z.F.); renrongping2026@163.com (R.R.);; 2 Hongdian Township Comprehensive Support and Technical Service Center, Wenshan Zhuang and Miao Autonomous Prefecture 663004, China; 3 School of Life Sciences, Taizhou University, Linhai 317000, China

**Keywords:** red-flowered strawberry, petal senescence, reactive oxygen species, ethylene, gene expression

## Abstract

Red-flowered strawberry possesses both ornamental and edible value, but its short single-flower lifespan severely limits its ornamental potential. In this study, petals of the red-flowered strawberry cultivar ‘140’ were collected at five developmental stages (large bud, half-bloom, full-bloom, initial withering, and withered) to systematically analyze the senescence process from morphological characteristics, physiological, and ethylene-related gene expression perspectives. The results showed that the epidermal cell breakage rate increased continuously during petal senescence, with lower epidermal cells consistently exhibiting higher breakage rates than the upper epidermal cells. Moisture content decreased progressively, while relative electrolyte leakage, malondialdehyde, hydrogen peroxide, and superoxide anion contents increased continuously. Superoxide dismutase, peroxidase, and catalase activities, as well as glutathione content, exhibited unimodal responses, peaking at different stages. Both ethylene and abscisic acid contents increased and then decreased, with ethylene showing greater amplitude and faster rate of change. The ethylene biosynthesis gene *FaACO1* and signaling genes *FaETR1*, *FaETR2*, *FaEIN2*, *FaEIN7*, *FaERF13*, and *FaERF118* were significantly upregulated at full-bloom or initial withering stages, with *FaERF118* showing the highest and continuously increasing expression. These findings indicate that water loss, membrane lipid peroxidation, and reactive oxygen species accumulation synergistically drive petal senescence, with the ethylene signaling pathway playing a key regulatory role.

## 1. Introduction

Strawberries (*Fragaria × ananassa* Duch.) are perennial herbaceous plants, belonging to the genus *Fragaria* in the Rosaceae family, and are widely cultivated worldwide with significant economic value. The vast majority of *Fragaria* species produce white flowers. For a long time, their fruits have been the primary focus of utilization. However, living standards have been rising. Consumer demands have become more diversified. As a result, red-flowered strawberries have gradually attracted the attention of breeders and consumers. These strawberries offer both ornamental and edible value.

Red-flowered strawberries are a new type of strawberry obtained by introducing red-flower genes into cultivated strawberries through distant hybridization. Their petals range in color from pink to deep red. Their green leaves, red flowers, and red fruits all possess ornamental value [1]. In 1989, Ellis crossed a white-flowered octoploid cultivated strawberry with a red-flowered hexaploid European red cinquefoil (*Potentilla palustris)* to develop the first pink-flowered strawberry variety ‘Pink Panda’ [2,3]. Since then, breeders have focused on several traits including flower color, flower form, disease resistance, and fruit quality, and have successively developed many new varieties, such as ‘Toscana’ and the ‘Summer Breeze’ series—‘Red-Pink Beauty’, ‘Pink Beauty’, ‘Charming Beauty’, ‘Purple-Gold Pink Jade’, and ‘Purple-Gold Red’—and the seed-propagated ‘Ruby Ann’ and ‘Roman’ [3,4]. Breeding efforts in China started relatively late [2]. Previous studies have also investigated the accumulation patterns of anthocyanins during petal development and the differential expression of related genes in red-flowered strawberries [5], as well as the phenotypic and genetic diversity of ornamental strawberry accessions [6]. Different red-flowered strawberry varieties exhibit rich diversity in flower color, flowering period, plant vigor, and stress tolerance [1]. However, the flowering period of individual flowers is generally short across all varieties. This severely limits the full realization of their ornamental value.

The short flowering period of red-flowered strawberries is a critical constraint for their adoption in ornamental horticulture [1]. In floriculture, flower longevity directly determines the economic value and consumer appeal of ornamental plants [7]. Extended flower longevity not only enhances aesthetic enjoyment but also reduces production costs and postharvest losses, making it a key breeding target for ornamental crops [8]. Studies have demonstrated that petal senescence is the primary determinant of flower longevity, and understanding its regulatory mechanisms is therefore essential for developing strategies to prolong the ornamental display period [9]. In cut flowers such as rose, carnation, and chrysanthemum, substantial progress has been made in elucidating the molecular basis of petal senescence, leading to effective postharvest treatments and breeding strategies for extended vase life [10]. By contrast, the mechanisms governing petal senescence in red-flowered strawberries remain largely unexplored, representing a significant knowledge gap that hampers the genetic improvement of their ornamental traits. Bridging this gap is not only of fundamental scientific interest but also of practical importance for the horticultural industry, as it could facilitate the development of cultivars with extended flowering periods and improved landscape performance.

The duration of the individual flower’s blooming period is essentially determined by the petal senescence process. Petal senescence is a genetically controlled developmental process. It is active and orderly. It manifests through several characteristics. These include changes in flower color, petal wilting, and petal abscission [11,12]. Current research on the mechanisms of petal senescence primarily revolves around three theories. These are the free radical damage theory [13,14,15,16], the nutrient deficiency theory [17,18,19], and the hormonal regulation theory [8,20,21,22,23,24,25,26].

At the core of the free radical damage theory lies the imbalance of reactive oxygen species (ROS) metabolism. During petal senescence, large amounts of ROS, such as H_2_O_2_ and O_2_^−^, are generated within cells, attacking unsaturated fatty acids in cell membranes and inducing membrane lipid peroxidation, which disrupts membrane structure and function [12]. Malondialdehyde (MDA), a major product of membrane lipid peroxidation, is widely used as a key indicator of oxidative damage during petal senescence. MDA content increases progressively as senescence advances in various ornamental species, reflecting the extent of ROS-induced membrane injury. Conversely, treatments that delay senescence have been shown to reduce MDA accumulation and maintain membrane integrity [27,28,29]. To scavenge excess ROS, plants have evolved enzymatic and non-enzymatic antioxidant defense systems, including superoxide dismutase (SOD), peroxidase (POD), catalase (CAT), and glutathione (GSH). Studies have shown that antioxidant enzyme activities increase during flower opening to eliminate ROS but decline significantly during senescence, leading to ROS accumulation and oxidative damage [30,31,32]. In addition, osmotic regulators such as proline, soluble sugars, and soluble proteins play important roles in maintaining osmotic balance and alleviating oxidative stress in petal cells. Exogenous proline treatment has been demonstrated to effectively alleviate oxidative stress in petal tissues and delay cut-flower senescence. Meanwhile, the decline in soluble sugar and soluble protein contents is a well-recognized indicator of the onset of senescence in plant organs.

In the context of hormonal regulation, ethylene is a key regulator of petal senescence, particularly in ethylene-sensitive species such as those in the Rosaceae family. Ethylene biosynthesis is controlled by two rate-limiting enzymes—ACC synthase (ACS) and ACC oxidase (ACO). In rose, ethylene accelerates petal senescence by inducing the F-box protein RhSAF, which promotes the degradation of the GA receptor RhGID1 [21]. The ethylene response factor RhERF113 modulates cytokinin content to regulate petal senescence [22], and the transcription factor RhMYB108 integrates ethylene and jasmonate signals to regulate senescence onset [23]. In carnation, DcWRKY33 promotes petal senescence by activating genes involved in ethylene (ET) and abscisic acid (ABA) biosynthesis, as well as ROS accumulation [24]. Moreover, ethylene signaling activates downstream senescence-associated genes through the EIN2-EILs cascade [10]. Recently, gene-editing technologies have been applied to regulate flower senescence, with targeted knockout of ethylene perception and signaling genes shown to significantly delay petal senescence. The latest studies have also revealed that the E3 ubiquitin ligase RED and its E2 partner UBC32 modulate ethylene responses by mediating the degradation of the ethylene receptor ETR3 through the ERAD pathway [33]. In strawberry, the expression of ethylene-related genes, including *FaACS*, *FaACO1*, *FaACO2*, *FaETR1*, *FaETR2*, *FaEIN2*, and *FaEIN7*, has been confirmed to be associated with flower organ development and senescence [34,35]. Collectively, these studies provide a fundamental basis for understanding the molecular mechanisms by which ethylene regulates petal senescence.

Most of these studies have focused on model flowers such as roses, carnations, and petunias [8,36]. However, no systematic studies on petal senescence in red-flowered strawberries have been reported to date. Red-flowered strawberries represent a novel ornamental plant.

A previous comprehensive evaluation of the ornamental qualities of 18 strawberry accessions was conducted by our research group. The evaluation indicated that the red-flowered strawberry variety ‘140’ had the highest overall score. It is well suited for home cultivation. However, its individual flower lifespan is only 2–3 days. During senescence, the petals become paler in color, lose their luster, soften in texture, and gradually wilt and curl. These changes severely limit its ornamental value. Therefore, this study used the red-flowered strawberry ‘140’ as the experimental material and defined five developmental stages: the large bud stage, half-open stage, full bloom stage, early senescence stage, and late senescence stage. The petal senescence process was systematically analyzed from three perspectives: morphological characteristics, physiological traits, and ethylene-related gene expression with the aims of elucidating the patterns of morphological, physiological, and molecular changes during petal senescence, preliminarily clarifying the potential role of ethylene in this process, providing a theoretical basis for extending the lifespan and enhancing ornamental value, and offering a reference for research on the mechanisms of petal senescence in ornamental plants.

## 2. Results

### 2.1. Petal Epidermal Cells of Red-Flowered Strawberries at Different Stages

**Figure 1:**
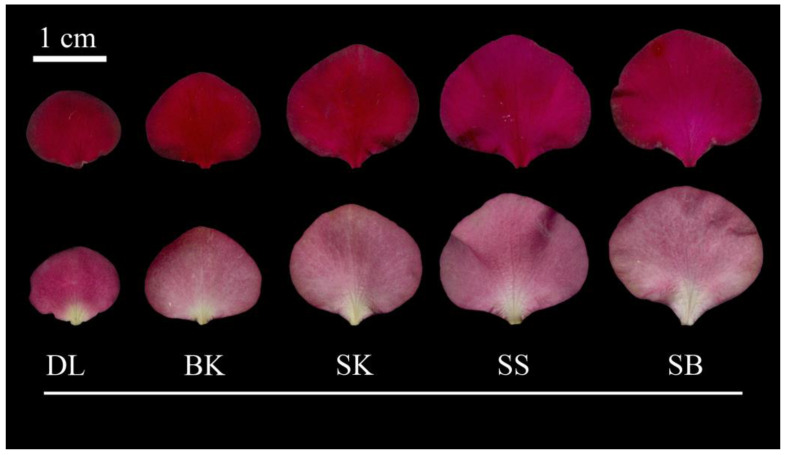
Petal at different periods. DL: large bud stage, BK: half-blooming stage, SK: full-blooming stage, SS: initial withering stage, and SB: withered stage.

The phenotypic characteristics of the petals of the red-flowered strawberry variety ‘140’ at different developmental stages were observed (Figure 1). During the opening stage (from the large bud stage to full bloom), the petal diameter gradually increased. The flower color remained vibrant. No obvious signs of senescence were observed. Upon entering the early senescence stage, the petal color noticeably faded. Surface luster diminished. The texture softened. Wilting occurred. Wrinkles gradually deepened. The petal margins began to curl. By the late senescence stage, more than half of the petal area exhibited irreversible wilting or shedding. This resulted in a significant loss of overall ornamental value.

**Figure 2:**
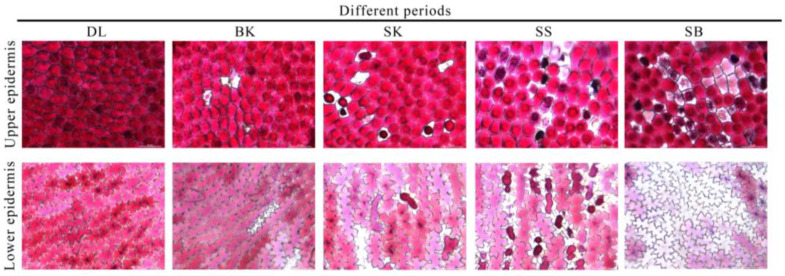
Microscopic observation of upper and lower epidermal cells of petals of red-flowered strawberry ‘140’ at different periods. DL: large bud stage, BK: half-blooming stage, SK: full-blooming stage, SS: initial withering stage, and SB: withered stage.

The upper and lower epidermis of the petals were prepared using the temporary water-mount method. Cell morphology was observed under a light microscope. The results are shown in Figure 2. During the large bud stage, epidermal cells were tightly packed. Cell outlines were clear. Cell color distribution was uniform. Virtually no cell damage was observed. During the half-open and full-bloom stages, cell color gradually lightened. The cell damage rate increased slightly. However, most cells retained their structural integrity. Upon entering the early senescence stage, a large number of epidermal cells exhibited damage and collapse. Cell outlines became blurred. Cell contents exuded from the cells. By the late senescence stage, the cell damage rate rose further. A large number of cells died. This indicated that the integrity of the cellular structure had been severely compromised. These results indicate two key points: first, cellular-level damage occurs prior to visible morphological wilting; second, the rate of cell rupture continues to increase as senescence progresses.

**Figure 3:**
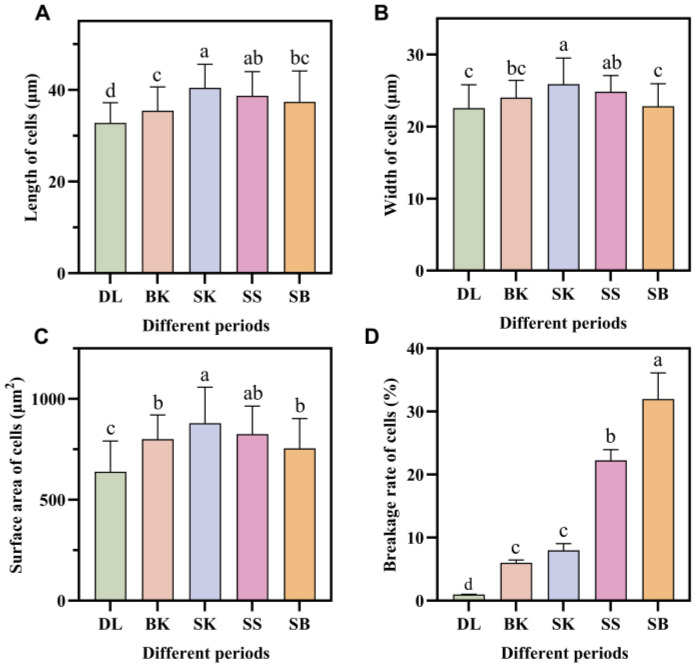
Variation in upper epidermal cell parameters on petals of red-flowered strawberry ‘140’ at different periods. (**A**) Change in length of upper epidermal cells, (**B**) change in width of upper epidermal cells, (**C**) change in surface area of upper epidermal cells, (**D**) change in breakage rate of upper epidermal cells. DL: large bud stage, BK: half bloom stage, SK: full bloom stage, SS: onset of decay stage, SB: decay stage. Different letters indicate significant difference at the 0.05 significance level.

During the development of the petals of the red-flowered strawberry ‘140’ from the large bud stage to the senescence stage, the length, width, and surface area of the upper epidermal cells all showed a trend of first increasing and then decreasing. All three parameters reached their maximum values at full bloom. Specifically, the length of the upper epidermal cells ranged from 32.80 to 40.43 μm (Figure 3A). The width ranged from 22.56 to 25.88 μm (Figure 3B). The surface area ranged from 754.91 to 879.61 μm^2^ (Figure 3C). The damage rate of upper epidermal cells showed a continuous upward trend as the senescence process progressed. It remained at a relatively low level from the large bud stage to the full bloom stage. However, it rose significantly after the onset of senescence. The overall range was from 0.94% to 37.28% (Figure 3D).

**Figure 4:**
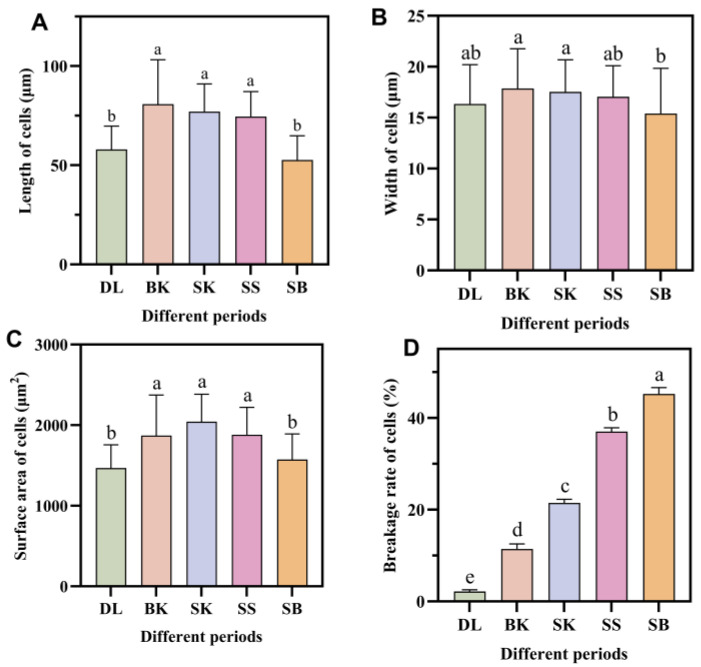
Variation in lower epidermal cell parameters on petals of red-flowered strawberry ‘140’ at different periods. (**A**) Change in length of lower epidermal cells, (**B**) change in width of lower epidermal cells, (**C**) change in surface area of lower epidermal cells, (**D**) change in breakage rate of lower epidermal cells. DL: large bud stage, BK: half-blooming stage, SK: full-blooming stage, SS: initial withering stage, and SB: withered stage. Different letters indicate significant difference at the 0.05 significance level.

During the development of the petals of the red-flowered strawberry cultivar ‘140’, from the large bud stage to the senescence stage, the length, width, and surface area of the lower epidermal cells all showed a trend of first increasing and then decreasing. Cell length and width reached their maximum values at the half-open stage. The cell length ranged from 52.63 to 80.83 μm (Figure 4A). The cell width ranged from 15.40 to 17.87 μm (Figure 4B). Cell surface area peaked at the full-bloom stage. It ranged from 1469.26 to 2043.71 μm^2^ (Figure 4C). The rupture rate of lower epidermal cells showed a continuous upward trend as senescence progressed. It ranged from 1.80% to 46.23% (Figure 4D). Throughout the senescence process, the rupture rate of lower epidermal cells remained consistently higher than that of upper epidermal cells.

### 2.2. Petal Color at Different Developmental Stages of Red-Flowered Strawberries

**Figure 5:**
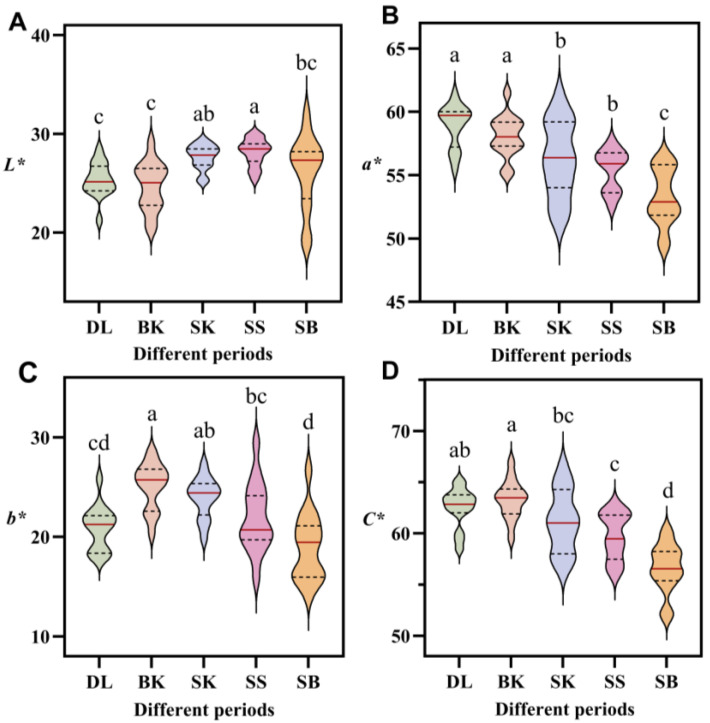
Petal changes in flower color values of red-flowered strawberry ‘140’ at different periods. (**A**) L* value (lightness): indicating the degree of lightness of the color; (**B**) a* value (redness/greenness): a positive value represents the degree of redness, the larger the value, the more reddish; (**C**) b* value (yellowness or blueness): a positive value represents the degree of yellowness, the larger the value, the more yellowish; (**D**) C* value (chroma): describing the vibrancy of the color. Same as follows. DL: large bud stage, BK: half-blooming stage, SK: full-blooming stage, SS: initial withering stage, and SB: withered stage. Different letters indicate significant difference at the 0.05 significance level.

An analysis of the colorimetric values of the petals of the red-flowered strawberry variety ‘140’ at five developmental stages is presented in Figure 5. As the petals developed from the large bud stage to the senescence stage, the L* value (luminance), b* value (yellow-blue hue), and C* value (chromaticity) all exhibited a trend of first increasing and then decreasing. Specifically, the L* value ranged from 19.10 to 32.04. It peaked at the onset of wilting. It reached its lowest point at the half-open stage (Figure 5A). The b* value ranged from 14.23 to 29.45. It peaked at the half-open stage. It reached its lowest point at the wilting stage (Figure 5C). The C* value ranged from 56.62 to 63.31. It peaked during the half-open stage. It reached its lowest point during the decline stage (Figure 5D). The a* value (redness), however, showed a continuous downward trend throughout the development process. It ranged from 53.26 to 58.92. The highest value was during the large bud stage. The lowest was during the decline stage (Figure 5B).

**Figure 6:**
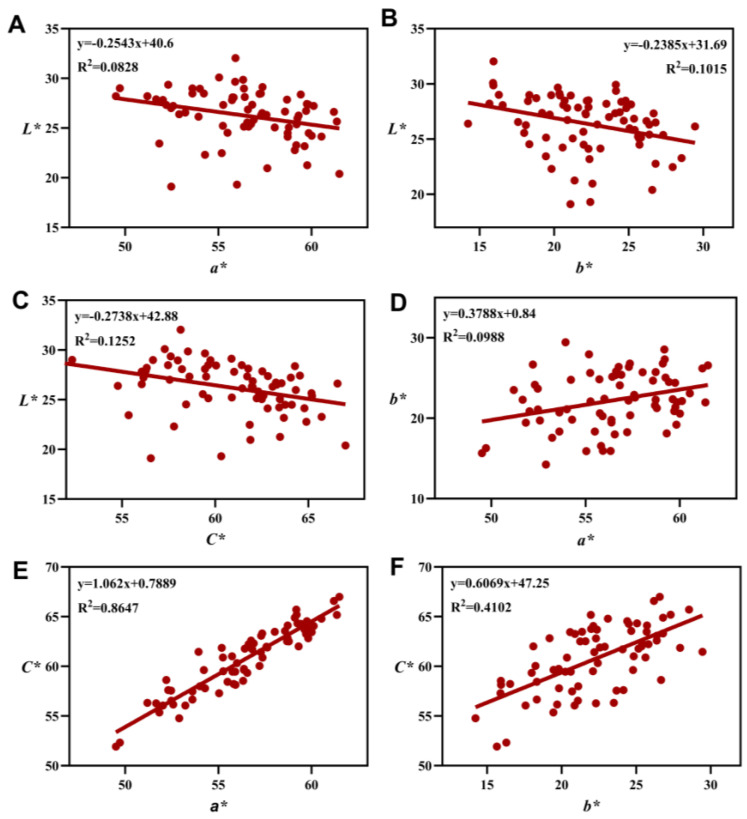
Correlation between petals L*, a*, b* and C* of red-flowered strawberry ‘140’. (**A**) Correlation between lightness (L*) and redness (a*); (**B**) correlation between lightness (L*) and yellowness (b*); (**C**) correlation between lightness (L*) and chroma (C*); (**D**) correlation between redness (a*) and yellowness (b*); (**E**) correlation between redness (a*) and chroma (C*); (**F**) correlation between yellowness (b*) and chroma (C*).

A correlation analysis was performed on the *L*, *a*, *b*, and *C* of ‘140’ petals. The results are shown in Figure 6. There was a highly significant positive correlation between a* and C* (R^2^ = 0.8647, *p* < 0.001) (Figure 6E). There was also a positive correlation between b* and C* (Figure 6F). This indicates that the combined contribution of petal redness and yellowness determines the level of chroma. There were no significant correlations between L* and *a*, *b*, or *C*. There was also no significant correlation between a* and b* (Figure 6A–D). This indicates that changes in lightness and color saturation are relatively independent.

### 2.3. Anthocyanin Content in Petals of Red-Flowered Strawberries at Different Developmental Stages

**Figure 7:**
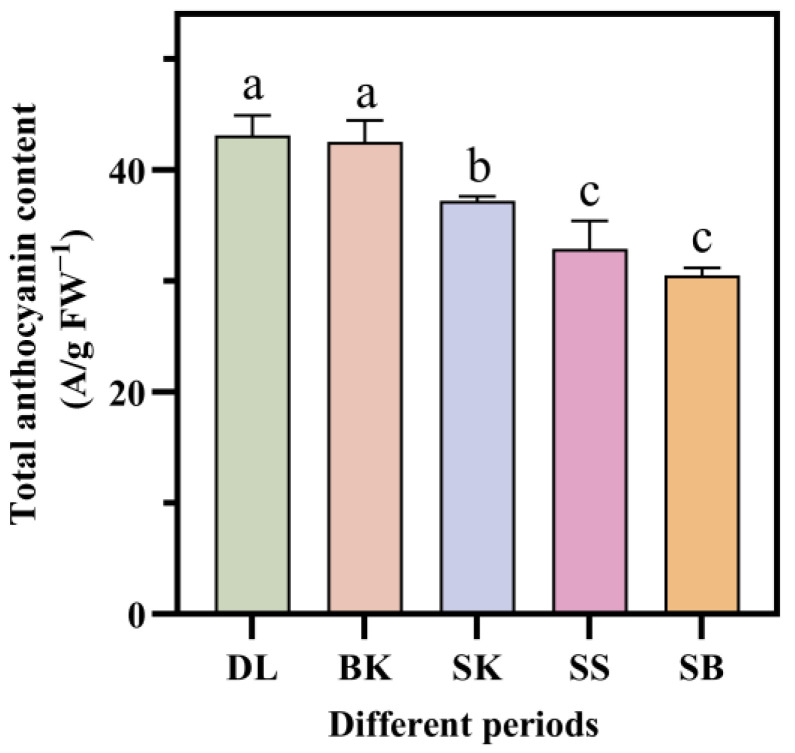
Total anthocyanin content of petals of red-flowered strawberry ‘140’ at different periods. DL: large bud stage, BK: half-blooming stage, SK: full-blooming stage, SS: initial withering stage, and SB: withered stage. Different letters indicate significant difference at the 0.05 significance level.

The total anthocyanin content in the petals of the red-flowered strawberry variety ‘140’ was measured at five developmental stages. The results are shown in Figure 7. As the petals developed from the large bud stage to the senescence stage, the total anthocyanin content showed a gradual decline. The content was highest during the large bud stage, decreased slightly during the half-open stage, although the decline was minor. Upon entering the full bloom stage, anthocyanin content decreased significantly. The decline was more pronounced compared to the half-open stage. Anthocyanin content continued to decline during the early senescence stage. It also continued to decline during the senescence stage. It reached its lowest level throughout the entire developmental process during the senescence stage.

### 2.4. Changes in Moisture Content, Relative Electrical Conductivity, and MDA Levels in the Petals of Red-Flowered Strawberries at Different Developmental Stages

**Figure 8:**
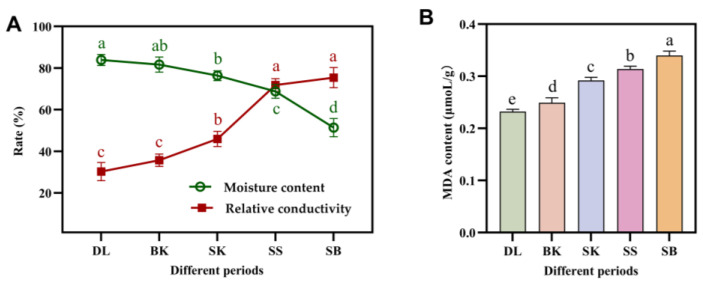
Changes in moisture content, relative conductivity and MDA content of petals of red-flowered strawberry ‘140’ at different periods. (**A**) moisture content and relative conductivity; (**B**) MDA content. DL: large bud stage, BK: half-blooming stage, SK: full-blooming stage, SS: initial withering stage, and SB: withered stage. Different letters indicate significant difference at the 0.05 significance level.

The moisture content of petals from the ‘140’ variety of red-flowered strawberries was measured at five developmental stages. The results are shown in Figure 8A. As the petals developed from the large bud stage to the senescence stage, the moisture content showed a continuous downward trend. It ranged from 51.37% to 83.86%. Specifically, moisture content was highest during the large bud stage (83.86%). It decreased slightly during the half-open stage (81.63%). It dropped to 76.32% during the full-bloom stage. Upon entering the early senescence stage, moisture content decreased significantly to 68.76%, and further declined to 51.37% during the senescence stage, indicating a continuous loss of water from the petals during the senescence process. The trends in relative electrolyte leakage rate and MDA content are shown in Figure 8B. During petal development, the relative electrolyte leakage rate exhibited a continuous upward trend. It ranged from 30.25% to 75.21%. MDA content also showed a continuous upward trend. It ranged from 0.23 to 0.34 μmol/L.

During the large bud stage, both the relative electrolyte leakage rate and MDA content were at their lowest levels (30.25%, 0.23 μmol/L). This indicates good membrane integrity at this stage. At the half-open stage, both indicators rose slightly (35.68%, 0.25 μmol/L). Cell membrane integrity remained relatively good. During the full-bloom stage, the relative electrolyte leakage rate rose to 45.93%. MDA content increased to 0.29 μmol/L. This indicates that cell membrane permeability had begun to increase. At the onset of senescence, both indicators rose significantly (71.85%, 0.31 μmol/L). This suggests that cell membrane integrity had been markedly compromised. During the senescence stage, both indicators reached their peak values (75.41%, 0.34 μmol/L). This indicates severe damage to the cell membrane structure. These results demonstrate that during the senescence process of the petals of the ‘140’ strawberry variety, continuous water loss, massive electrolyte exudation, and accelerated membrane lipid peroxidation occur simultaneously. These changes collectively lead to the functional decline of the cell membrane system.

### 2.5. H_2_O_2_ and O_2_^−^ Levels in the Petals of Red-Flowered Strawberries at Different Developmental Stages

**Figure 9:**
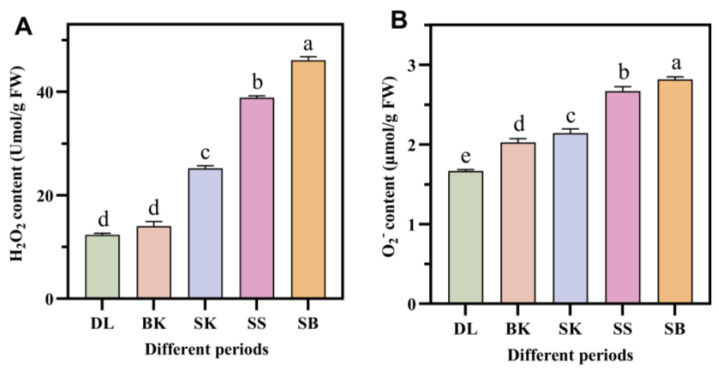
Changes in H_2_O_2_ and O_2_^−^ content of petals of red-flowered strawberry ‘140’ at different periods. (**A**) H_2_O_2_ content; (**B**) O_2_^−^ content. DL: large bud stage, BK: half-blooming stage, SK: full-blooming stage, SS: initial withering stage, and SB: withered stage. Different letters indicate significant difference at the 0.05 significance level.

The H_2_O_2_ and O_2_^−^ levels in the petals of the red-flowered strawberry variety ‘140’ were measured at five developmental stages. The results are shown in Figure 9. As the petals developed from the large bud stage to the senescence stage, both H_2_O_2_ and O_2_^−^ concentrations exhibited a continuous upward trend. H_2_O_2_ concentrations remained at relatively low levels during the large bud and half-open stages. They rose significantly starting from the full bloom stage. They remained at high levels throughout the early senescence and senescence stages. They ranged from 9.52 to 46.08 μmol/g FW (Figure 9A). O_2_^−^ levels showed a stepwise increase during the senescence process. They ranged from 1.67 to 2.82 μmol/g FW (Figure 9B). These results indicate that ROS accumulate continuously during the senescence of the petals of the red-flowered strawberry variety ‘140’.

### 2.6. SOD, POD, and CAT Activities and GSH Content in Petals of the Red-Flowered Strawberry at Different Developmental Stages

**Figure 10:**
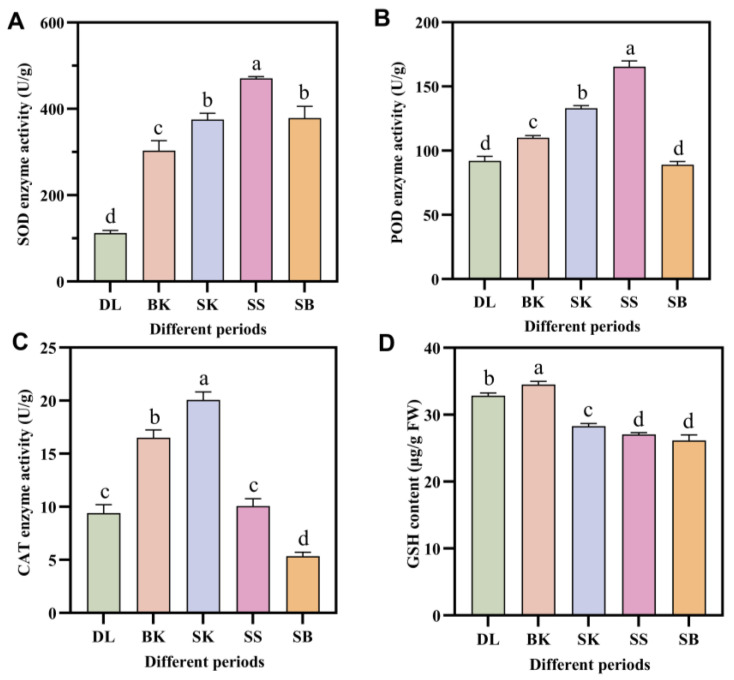
Changes in SOD, POD, CAT enzyme activity and GSH content in petals of red-flowered strawberry ‘140’ at different periods. (**A**) SOD activity; (**B**) POD activity; (**C**) CAT activity; (**D**) GSH content. DL: large bud stage, BK: half-blooming stage, SK: full-blooming stage, SS: initial withering stage, and SB: withered stage. Different letters indicate significant difference at the 0.05 significance level.

The antioxidant enzyme activities and levels of non-enzymatic antioxidants in the petals of the red-flowered strawberry variety ‘140’ were measured at five developmental stages. The results are shown in Figure 10. As the petals developed from the large bud stage to the senescence stage, the activities of SOD, POD, and CAT, as well as GSH content, all exhibited a trend of first increasing and then decreasing. However, the timing at which each indicator reached its peak varied.

SOD and POD activities followed similar trends, both gradually increasing from the large bud stage, peaking at the onset of senescence, and then declining. SOD activity ranged from 112.20 to 470.56 U/g FW (Figure 10A). POD activity ranged from 89.00 to 165.33 U/mL FW (Figure 10B). CAT activity peaked during the full-bloom stage. It declined significantly after the onset of senescence. It ranged from 5.35 to 20.08 U/g FW (Figure 10C). GSH content peaked during the semi-open stage. It then continued to decline. It ranged from 13.22 to 34.51 μg/g FW (Figure 10D). These results indicate that SOD and POD play the primary roles in scavenging oxygen free radicals during the late senescence stage of the petals of the red-flowered strawberry variety ‘140’.

### 2.7. Proline, Soluble Sugars and Proteins in the Petals of the Red-Flowered Strawberry at Different Developmental Stages

**Figure 11:**
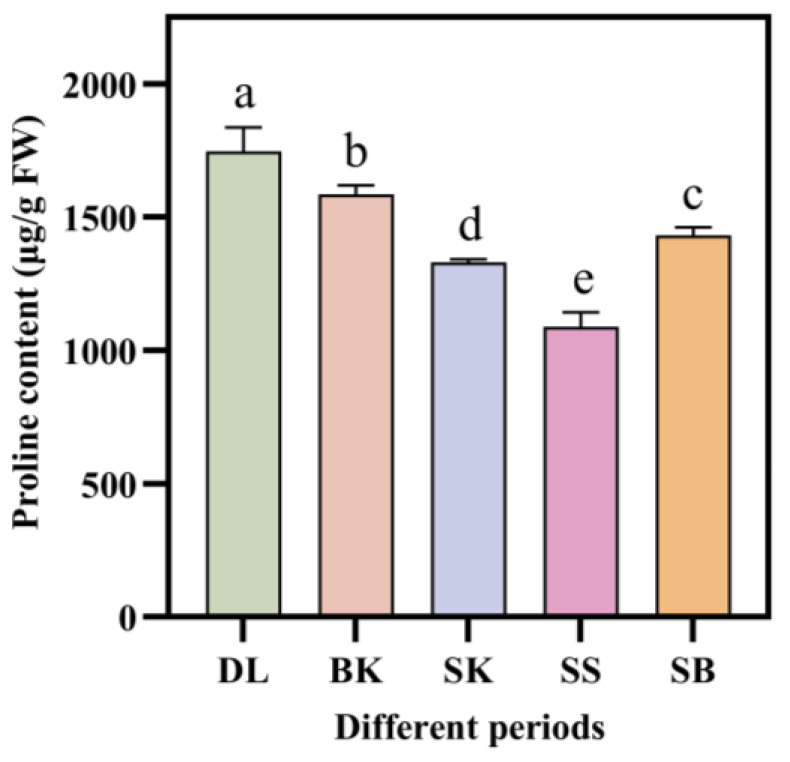
Changes in proline of petals of red-flowered strawberry ‘140’ at different periods. DL: large bud stage, BK: half-blooming stage, SK: full-blooming stage, SS: initial withering stage, and SB: withered stage. Different letters indicate significant difference at the 0.05 significance level.

**Figure 12:**
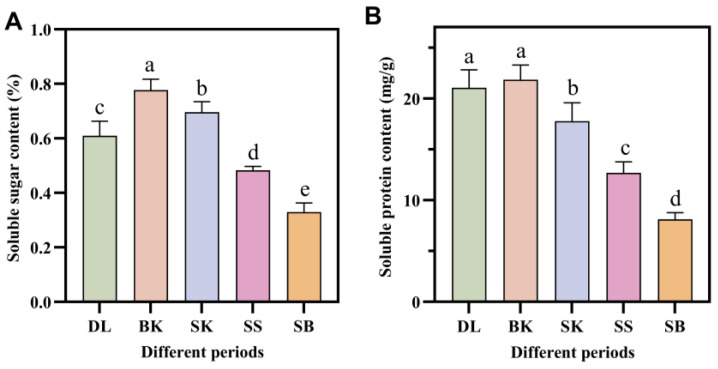
Changes in soluble sugar and soluble protein content of petals of red-flowered strawberry ‘140’ at different periods. (**A**) soluble sugar content; (**B**) soluble protein content. DL: large bud stage, BK: half-blooming stage, SK: full-blooming stage, SS: initial withering stage, and SB: withered stage. Different letters indicate significant difference at the 0.05 significance level.

The concentrations of osmoregulatory substances in the petals of the red-flowered strawberry variety ‘140’ were measured at five developmental stages. The results are shown in Figure 11 and Figure 12.

Proline content exhibited a trend of first decreasing and then increasing during petal development (Figure 11). Proline content was highest during the large bud stage (1746.37 μg/g) and then continued to decline as development progressed, reaching a minimum (1088.21 μg/g) at the onset of senescence, before rebounding significantly during the senescence stage (1431.83 μg/g).

The trends in soluble sugar and soluble protein content were generally consistent (Figure 12). Soluble sugar content showed an upward trend from the large bud stage (0.61%) to the half-open stage (0.78%). It then gradually decreased after the half-open stage (Figure 12A). Soluble protein content remained at a relatively high level during the large bud stage (21.04 mg/g). It also remained high during the half-open stage (21.83 mg/g). However, it began to decline continuously starting from the full-bloom stage (17.77 mg/g) (Figure 12B).

These results indicate that soluble sugar and soluble protein contents increase during the early stages of petal opening. This provides material and energy reserves for petal growth. They then gradually decrease upon entering the senescence stage.

### 2.8. Ethylene and ABA Content in Petals of Red-Flowered Strawberries at Different Stages

**Figure 13:**
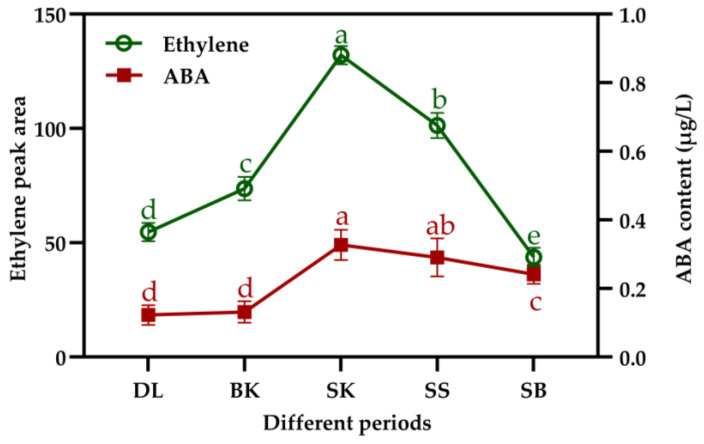
Ethylene and ABA changes in petals of red-flowered strawberry ‘140’ at different periods. DL: large bud stage, BK: half-blooming stage, SK: full-blooming stage, SS: initial withering stage, and SB: withered stage. Different letters indicate significant difference at the 0.05 significance level.

Ethylene release rates and ABA content in the petals of the red-flowered strawberry variety ‘140’ were measured at five developmental stages. The results are shown in Figure 13. Both the ethylene peak area and ABA content exhibited a trend of first increasing and then decreasing during petal development. However, the magnitude of these changes differed. The ethylene peak area began to rise from the large bud stage, increased significantly at the half-open stage, reached a peak (132) at the full-bloom stage, and then gradually declined.

ABA content remained relatively stable from the large bud stage to the half-open stage. It then rose significantly at the full bloom stage. It gradually decreased afterwards. The magnitude of the increase in ethylene peak area was significantly greater than that of ABA. The rate of change in ethylene peak area was also significantly greater than that of ABA. This indicates that the ‘140’ red-flowered strawberry is more sensitive to ethylene in response to petal senescence.

### 2.9. Correlation Analysis of Physiological Indicators

**Figure 14:**
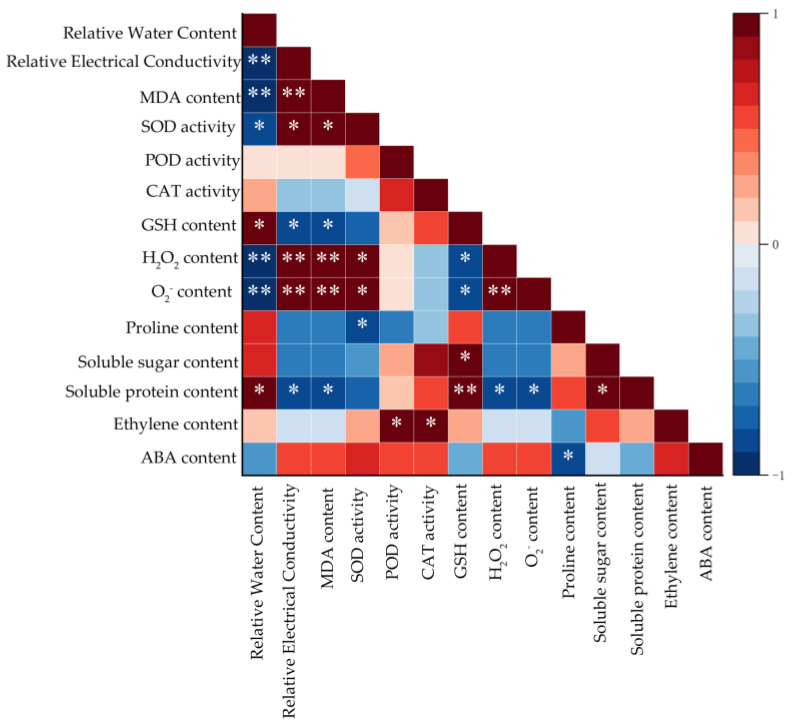
Correlation analysis between physiological indicators related to petal senescence. * Indicates significant difference at the 0.05 level, and ** indicates significant difference at the 0.01 level.

A correlation analysis was conducted on physiological indicators related to petal senescence in the ‘140’ strawberry variety. The results are shown in Figure 14. Moisture content exhibited a highly significant negative correlation with relative electrolyte leakage rate, MDA content, H_2_O_2_ content, and O_2_^−^ content. Moisture content showed a significant positive correlation with GSH content and soluble protein content. Relative electrolyte leakage rate, MDA content, H_2_O_2_ content, and O_2_^−^ content all exhibited highly significant positive correlations with one another. These results indicate that water loss, exacerbated membrane lipid peroxidation, and ROS accumulation are interrelated. They collectively promote the progression of petal senescence in the ‘140’ red-flowered strawberry.

### 2.10. qRT-PCR Analysis of Genes Involved in Ethylene Synthesis and Signal Transduction

**Figure 15:**
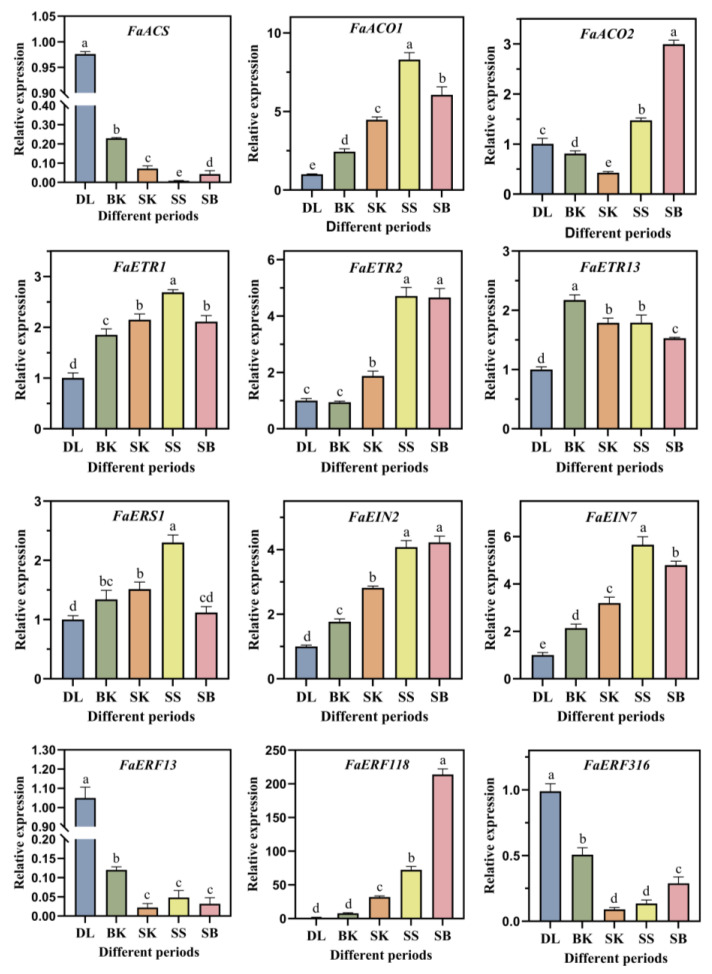
Changes in the expression of ethylene synthesis and signaling-related genes in petals of red-flowered strawberry ‘140’ at different periods. DL: large bud stage, BK: half-blooming stage, SK: full-blooming stage, SS: initial withering stage, and SB: withered stage. Different letters indicate significant difference at the 0.05 significance level.

To investigate the potential role of ethylene at the molecular level, qRT-PCR was used to detect ethylene-related genes in petals at five developmental stages. The genes included ethylene synthesis-related genes and ethylene signal transduction-related genes. The results are shown in Figure 15.

Based on their expression trends, the 12 genes can be classified into four categories. The first category includes *FaETR2*, *FaEIN2*, and *FaERF118*. Their expression levels continuously increased as senescence progressed. This category represents a continuously increasing trend. The second category includes *FaACO1*, *FaETR1*, *FaETR13*, *FaERS1*, and *FaEIN7*. These five genes showed an upward trend from the large bud stage to the early senescence stage. They then gradually declined. Among these, *FaETR13* reached its peak at the half-open stage. The remaining four genes all peaked at the onset of senescence. This category represents a trend of first increasing and then decreasing. The third category includes *FaACO2* and *FaERF316*. Their expression levels continuously decreased from the large bud stage to the full bloom stage. They then rebounded after full bloom. This category represents a trend of first decreasing and then increasing. The fourth category includes *FaACS* and *FaERF13*. Their expression levels showed a gradual downward trend throughout the entire developmental process. This category represents a continuous decline.

These results indicate that the expression of genes related to the ethylene signaling pathway exhibits diverse regulatory patterns during the petal senescence process of the red-flowered strawberry ‘140’.

## 3. Discussion

Petal senescence is a genetically regulated process. It is a form of programmed cell death. This study systematically analyzed the senescence patterns of the petals of the red-flowered strawberry variety ‘140’. The analysis covered the period from the large bud stage to the withering stage. It was conducted at the morphological, physiological, and molecular levels. The study revealed the main characteristics of petal senescence in this variety. It also revealed the potential role of ethylene in this process.

### 3.1. Cytological Changes and Flower Color Fading

In this study, both upper and lower epidermal cells first expanded and then contracted during the opening process, and a significant increase in the cell rupture rate preceded visible wilting, suggesting that damage at the cellular level is an early event in petal senescence. The cell rupture rate in lower epidermal cells remained consistently higher than that in upper epidermal cells. This may be related to their larger cell volume and thinner cell walls. Farooq et al. [9] pointed out that petal senescence involves a series of biochemical changes. These include reactive oxygen species (ROS) production, membrane degradation, and nucleic acid and protein degradation. Petal senescence is a typical form of programmed cell death.

Programmed cell death during petal senescence is characterized by gradual cytoplasm condensation, organelle degradation, and ultimately vacuole rupture, which releases hydrolytic enzymes and causes the rapid breakdown of cellular contents [7,11]. The progressive increase in epidermal cell breakage observed in this study, particularly the higher susceptibility of lower epidermal cells, is consistent with these PCD features. The temporal coincidence between vacuolar rupture and anthocyanin degradation further supports the notion that loss of vacuolar compartmentalization is a key event triggering pigment catabolism during senescence [37], consistent with the characteristics of programmed cell death described above.

Anthocyanin content and the a* value (redness) exhibited a continuous downward trend. In contrast, the L value, b* value, and *C* value followed a bell-shaped curve. They first rose and then fell. This suggests that ‘140’ underwent complex pigment metabolic reorganization during the senescence process. In the correlation analysis, a highly significant positive correlation was found between a* and *C* (R^2^ = 0.8647, *p* < 0.001). This confirmed that anthocyanins are the dominant factor determining the chroma of ‘140’ petals. The degradation of anthocyanins closely coincided temporally with the increase in cell rupture rate. As vacuoles ruptured, anthocyanins lost the protection of the acidic environment within the vacuoles. This led to accelerated degradation. A study by Zhong et al. [37] demonstrated that the degradation of anthocyanins during the postharvest senescence of strawberries is closely related to the disruption of cellular structure.

### 3.2. Water Loss and Membrane Lipid Peroxidation

In this study, moisture content decreased continuously from 83.86% to 51.37%. The relative electrolyte leakage rate increased from 30.25% to 75.21%. MDA content rose from 0.23 μmol/L to 0.34 μmol/L. Correlation analysis confirmed the existence of a positive feedback loop among water loss, increased membrane lipid peroxidation, and ROS accumulation. Zeng et al. [38] found that the vase life of cut roses is closely related to water-related parameters of the flower stems. Water imbalance is a key factor leading to accelerated petal senescence. The simultaneous exacerbation of water loss and membrane damage was observed in this study. This further supports the view that water imbalance promotes senescence by intensifying oxidative stress.

### 3.3. Accumulation of Reactive Oxygen Species and Antioxidant Defense

The levels of H_2_O_2_ and O_2_^−^ continue to rise during the aging process. In contrast, the activities of SOD, POD, and CAT, as well as GSH levels, exhibit a ‘first rise, then decline’ response pattern. There are significant differences in the timing of the peaks for these four components. GSH peaks during the semi-bloom stage. CAT peaks during the full bloom stage. SOD and POD peak during the early decline stage. This temporal difference suggests that antioxidant defense is a multilevel, phased dynamic process. Non-enzymatic antioxidants respond first. H_2_O_2_-scavenging enzymes follow. Superoxide anion-scavenging enzymes reach their maximum activity last.

Jiang et al. [39] found in roses that the *RhWRKY33a-RhPLATZ9* regulatory module delays petal senescence. It does so by inhibiting the rapid accumulation of ROS. The sustained high activity of SOD and POD during the late stages of senescence is a key downstream event of this regulatory module. Wang et al. [24] also found in carnations that *DcWRKY33* promotes petal senescence. It does so by activating genes related to ethylene and ABA biosynthesis. It also activates genes related to ROS accumulation. This reveals a regulatory network between hormones and ROS. In this study, SOD and POD activity declined somewhat after peaking in the early senescence stage, but remained relatively high during the late senescence stage, suggesting that the red-flowered strawberry variety ‘140’ may maintain the expression of specific antioxidant enzymes through similar transcriptional regulatory mechanisms to cope with oxidative stress in the late stages of senescence.

Beyond their direct scavenging function, ROS also act as signaling molecules that activate stress-responsive pathways [14,40]. The transient increase in antioxidant enzyme activities during flower opening may reflect a programmed priming response, while their subsequent decline marks the transition from active defense to passive deterioration [40].

### 3.4. Regulatory Role of Ethylene Signaling

Most Rosaceae plants are ethylene-sensitive. In this study, both the magnitude and rate of change in the peak area of ethylene were significantly greater than those of ABA. Several key genes in ethylene signaling pathways (*FaACO1*, *FaETR1*, *FaETR2*, *FaEIN2*, *FaEIN7*, *FaERF13*, *FaERF118*) were significantly upregulated during the full bloom or early senescence stages. This strongly suggests that petal senescence in ‘140’ is significantly influenced by ethylene. However, the magnitude of ethylene accumulation was greater than that of ABA, suggesting that ethylene may be a more prominent hormonal signal during senescence in this cultivar. Direct comparisons of hormone accumulation do not establish their relative biological importance, and the involvement of ABA should be further investigated through functional analyses. Lu et al. [21] found in roses that the ethylene-induced F-box protein RhSAF accelerates petal senescence. It does so by promoting the degradation of the GA receptor *RhGID1*. This reveals the molecular mechanism by which ethylene promotes senescence by antagonizing GA signaling.

In addition to ethylene, other hormones, such as cytokinins and gibberellins, are known to delay petal senescence, while jasmonates synergize with ethylene to promote it [8,20,26]. The relative contribution of these hormones to senescence in red-flowered strawberry remains unknown, as our study focused primarily on ethylene and ABA. The detection of other hormone signals and their pathway genes in future work would provide a more complete picture of the hormonal regulatory network.

The expression level of *FaERF118* continued to rise throughout the senescence process. It was the highest among the genes analyzed. This suggests that it may be a key downstream response factor in the ethylene signaling pathway during petal senescence in ‘140’. The continuous decline in *FaACS* expression from the large bud stage to full bloom appears contradictory. This decline is coupled with the concurrent increase in ethylene peak area.

However, as a rate-limiting enzyme, ACS activity is finely regulated by post-translational phosphorylation modifications and degradation. Furthermore, ethylene synthesis is synergistically regulated by other genes, such as *FaACO1*. Therefore, the transcriptional level of *FaACS* does not fully reflect the rate of ethylene synthesis. Farooq et al. [41] pointed out that manipulating genes related to ethylene biosynthesis or signal transduction has shown potential for extending flower lifespan. This provides a possible technical approach for regulating the ornamental period of the red-flowered strawberry.

In summary, this study systematically mapped the dynamic profile of petal senescence in the red-flowered strawberry ‘140’ at the morphological, physiological, and molecular levels. The ethylene signaling pathway plays a key regulatory role in petal senescence of ‘140’. However, its specific action points and downstream target genes still need to be confirmed through functional validation experiments.

### 3.5. Study Limitations and Future Perspectives

The present study is correlative in nature and does not establish a causal role for ethylene or ABA. The greater magnitude of ethylene accumulation compared to ABA does not necessarily indicate greater biological importance, as hormone accumulation levels may reflect differences in biosynthesis, turnover, or tissue distribution rather than signaling potency. Furthermore, we did not examine ABA biosynthesis or signaling genes, nor did we perform functional validation through exogenous hormone or inhibitor treatments. The involvement of other hormones, such as cytokinins, gibberellins, and jasmonates, also remains unexplored. Future studies employing integrated multi-omics approaches, combined with functional experiments such as exogenous ethylene inhibitor treatments, silencing or overexpression of ethylene signaling genes, and transcriptomic analyses, are needed to determine the relative contributions of these hormones and to systematically identify key regulatory networks governing petal senescence in red-flowered strawberry.

## 4. Materials and Methods

### 4.1. Plant Materials

The ‘140’ red-flowered strawberry was cultivated in a constant-temperature and constant-humidity incubator. The incubator was maintained at 20 °C during the day and 15 °C at night, with a relative humidity of 65%.

Petals from the ‘140’ red-flowered strawberry were collected at five different stages. Three independent biological replicates were performed for each developmental stage, with each replicate consisting of petals collected from three individual plants (one flower per plant). The stages were the large bud stage, half-open stage, full bloom stage, early decline stage, and wilting stage. These were used as experimental materials (Figure 1). Approximately 1 day passed between each stage. For each biological replicate, fresh petal samples were allocated as follows: three petals per flower were randomly selected for color measurement and physiological assays (including moisture content and electrical conductivity), and additional petal segments were used for microscopic observation of epidermal cells. The remaining samples were rapidly frozen in liquid nitrogen. They were ground into powder using a grinder and stored in a −80 °C freezer for future use. The different stages were defined as follows:

(1) Large bud stage (DL): Sepals are open; petals are closed and fully colored, exhibiting the deepest hue.
(2) Half-open stage (BK): Petals are half-open and extend above the sepals.
(3) Full bloom stage (SK): Sepals are spread flat, the flower is fully open, and it is at its most visually appealing stage.
(4) Early decline stage (SS): Petals begin to wilt and lose color, showing the first visible signs of senescence.
(5) Decline stage (SB): More than half of the petals have wilted or fallen off, with the flower losing most of its ornamental value.

### 4.2. Microscopic Observation of Petal Epidermal Cells

The procedure is as follows: Carefully peel the epidermis from the edge of the petal using fine tweezers. Completely detach the epidermal layer along the direction of the vascular bundles. Quickly spread it flat on a microscope slide with a drop of distilled water. Cover with a coverslip. This prepares a temporary water mount [42]. Observe under an optical microscope using a 40× objective lens. Select areas with clear cell morphology and uniform distribution. Photograph and record these areas.

### 4.3. Flower Color Measurement

Following the method described by Nishihara [43], we measured the color using a spectrophotometer YS3060, (Shenzhen 3nh Technology Co., Ltd., Shenzhen, China) with the aperture aligned with the center of the petal. The instrument parameters were as follows: standard illuminant D65, standard observer angle 10°, and measurement aperture 4 mm. Three petals were randomly selected from each flower for measurement, and the average values were calculated. The color parameters L*, a*, and b* were recorded, where L* represents lightness, with values ranging from 0 (black) to 100 (white); the a* value represents redness or greenness, with positive values indicating redness and negative values indicating greenness; the b* value represents yellowness or blueness, with positive values indicating yellowness and negative values indicating blueness; the chroma value C* is calculated using Equation (1).

(1) $$C^{*}=\sqrt{\left({\left(a^{*}\right)}^{2}+({b^{*})}^{2}\right)}$$

### 4.4. Determination of Total Anthocyanin Content

The total anthocyanin content in petals was determined using acid methanol extraction followed by spectrophotometry. We transferred 0.1 g of petal powder into a 10 mL centrifuge tube, added 5 mL of 1% hydrochloric acid in methanol solution (1:99, *v*/*v*), vortexed to mix, and extracted it at 4 °C in the dark for 24 h, shaking frequently during this period. After extraction, it was centrifuged at 4 °C and 12,000 r/min for 20 min, and we collected the supernatant. We measured the absorbance of the supernatant at wavelengths of 530 nm and 657 nm using a microplate reader. The relative total anthocyanin content was calculated using the formula A_530_ − 0.25 × A_657_ [44] to correct for the interference of chlorophyll and its degradation products on the absorbance at 530 nm. The content was expressed as absorbance units per gram fresh weight (A/g FW) after normalization against sample fresh weight. Each sample was tested in triplicate.

### 4.5. Determination of Moisture Content

After collecting petals at different stages, we immediately weighed their fresh mass (W_1_), then dried them in an oven at 80 °C until constant weight was reached, and weighed their dry mass (W_2_). The moisture content of the petals was calculated using the following formula:

(2) $$Moisture\;Content(\%)=\frac{W_{1}-W_{2}}{W_{1}}\times 100\%$$

### 4.6. Determination of Oxidative Damage Indicators

Hydrogen peroxide and superoxide anion content: Determined using the corresponding assay kit from Beijing Solabio Science &Technology Co., Ltd., Beijing, China (Cat. No. BC3595 for H_2_O_2_ and Cat. No. BC1295 for O_2_^−^), with specific procedures following the kit instructions.

Relative Electrolyte Leakage Rate: Determined using the boiling method [45]. Petal discs with a diameter of 1 cm were prepared using a punch. A 0.05 g petal disc was weighed and placed in 20 mL of ultrapure water and then incubated with shaking at 28 °C and 180 r/min for 30 min to measure the initial conductivity (R_1_). The sample was then heated in a boiling water bath for 30 min; after cooling, the total conductivity (R_2_) was measured. The relative electrolyte leakage rate was calculated using the following formula:

(3) $$Relative\;electrolyte\;leakage\;rate(\%)=\frac{R_{1}}{R_{2}}\times 100\%$$

Malondialdehyde (MDA) content: Determined using the thiobarbituric acid (TBA) method [46]. We transferred 2 mL of supernatant (V_1_) to a test tube, added 2 mL of 0.67% thiobarbituric acid solution, mixed thoroughly, and heated it in a boiling water bath for 30 min. After cooling in an ice bath, we centrifuged the mixture; the supernatant was the sample to be analyzed (V_2_). We measured the absorbance of the test solution at wavelengths of 450 nm, 532 nm, and 600 nm using a microplate reader; we performed three biological replicates for each sample. The MDA concentration and content were calculated using the following formulas:

(4) $$MDA\;content(µmol/g)=\frac{C\times V_{2}\times V}{m\times V_{1}\times 1000}$$

where *C* is the MDA concentration in the test solution (μmol/L); *V* is the total volume of the extract (mL); *V*_1_ is the volume of sample extract added to the test solution (mL); *V*_2_ is the total volume of the test solution (mL); *m* is the sample mass (g); and the factor 1000 is used to convert the volume unit from μL to mL.

### 4.7. Determination of Antioxidant Parameters

SOD, POD, and CAT enzyme activities, as well as GSH content, were determined using the SOD Activity Assay Kit (Cat. No. BC0175), POD Activity Assay Kit (Cat. No. BC0095), and CAT Activity Assay Kit (Cat. No. BC0205), respectively, from Beijing Solabio Technology Co., Ltd., along with the GSH Content Assay Kit (Cat. No. BC1175).

### 4.8. Determination of Osmoregulatory Substances

Proline was determined using the Pro Content Assay Kit from Beijing Solabio Technology Co., Ltd. (Cat. No. BC0290).

Soluble sugar content was determined using the anthrone-sulfuric acid colorimetric method [47]. We weighed 0.1 g of petal powder, transferred the sample to a 20 mL test tube in three stages using 10 mL of distilled water each time, sealed the tube, and extracted it in a boiling water bath for 30 min; we repeated this extraction process twice. We combined the extractions, filtered them into a 25 mL volumetric flask, and after repeatedly rinsing the test tubes and residue, made it up to volume.

We transferred 1 mL of the sample filtrate to a 10 mL test tube, added 0.5 mL of anthrone–ethyl acetate reagent followed by 5 mL of concentrated sulfuric acid, shook thoroughly, and immediately placed it in a boiling water bath for 1 min. After cooling to room temperature, we measured the absorbance at a wavelength of 630 nm. We repeated this procedure three times for each sample. We plotted a standard curve using sucrose as the standard. We determined the sugar content (C) in the sample from the standard curve and calculated the soluble sugar content using the following formula:

(5) $$Soluble\;sugar\;content(\%)=\frac{C\times V\times N}{a\times W\times 1000000}\times 100\%$$

where *C* is the sugar content (μg) determined from the standard curve; *V* is the volume of the extract (mL); *N* is the dilution factor; a is the volume of the sample solution used for determination (mL); W is the sample weight (g); and the factor 1,000,000 is used to convert μg to g.

The soluble protein content was determined using the Coomassie Brilliant Blue G-250 method (Bradford method) [48]. We transferred 0.2 g of petal powder into a 10 mL centrifuge tube, added 6 mL of phosphate buffer (pH 7.0), shook to mix thoroughly, and allowed it to stand at room temperature for 30 min to ensure complete extraction. It was centrifuged at 4 °C and 4000 r/min for 20 min; the supernatant was the sample solution. We transferred 0.2 mL of the sample solution to a test tube, added 0.8 mL of distilled water followed by 5 mL of Coomassie Brilliant Blue G-250 reagent, mixed thoroughly, let stand for 2 min, and then measured the absorbance at a wavelength of 595 nm. We plotted a standard curve using bovine serum albumin (BSA) as the standard. We determined the protein content (C) in the sample from the standard curve and calculated the soluble protein content using the following formula:

(6) $$Soluble\;protein\;content(mg/g)=\frac{C\times V_{t}}{V_{s}\times W_{t}\times 1000}$$

where C is the protein concentration (μg) determined from the standard curve. *V_T_* is the total volume of the extract (mL). *V_s_* is the sample volume added during the assay (mL); *W_t_* is the sample mass (g).

### 4.9. Determination of Plant Hormone Content

Ethylene release was determined by gas chromatography, following the method described by Cronje and Jonker [49]. Fresh petals (approximately 0.5 g) were sealed in 20 mL glass vials and incubated at 25 °C for 2 h in the dark. A 1 mL headspace gas sample was withdrawn using a gas-tight syringe and injected without split injection. The retention time was set to 5 min. The injection was repeated three times. Ethylene concentration was quantified using a standard curve prepared from ethylene standard gases at concentrations of 1, 10, 50, and 100 μL/L. Ethylene production was expressed as peak area (relative units) and was not converted to absolute concentration. The gas chromatography conditions were as follows: GC-2010Pro gas chromatograph (Shimadzu, Kyoto, Japan), SPH-300 hydrogen generator (Beijing Zhonghuipu Analysis Technology Institute, Beijing, China), and LBC-PLOTQ column (30 m × 0.53 mm × 20 μm, Agilent Technologies, Santa Clara, CA, USA). The column flow rate was 2 mL/min. The column temperature was 40 °C. The split ratio was 30:1. The detector was a hydrogen flame ionization detector (FID) at 250 °C. The H_2_ fuel flow rate was 40 mL/min. The combustion air flow rate was 450 mL/min. The carrier gas (N_2_) flow rate was 45 mL/min.

Abscisic acid (ABA) content was determined using a plant hormone ABA enzyme-linked immunosorbent assay (ELISA) kit (Jiangsu Enzyme Immuno Industry Co., Ltd., Yancheng, China). Specific procedures followed the kit instructions.

### 4.10. qRT-PCR Analysis of Genes Related to Ethylene Synthesis and Signal Transduction

Total RNA was extracted from petals at various developmental stages using the Polysaccharide and Polyphenol Plant Total RNA Extraction Kit (Centrifugation Column Type) from Tiangen BioTech (Beijing) Co., Ltd., Beijing, China. RNA concentration and purity were preliminarily assessed using a UV–visible spectrophotometer (OD260/280 and OD260/230 ratios, with ddH_2_O as the blank control), and RNA integrity was examined via 1.2% agarose gel electrophoresis. The extracted RNA was reverse-transcribed into cDNA using the ABScript Neo RT Master Mix for qPCR with a gDNA Remover reverse transcription kit from Wuhan Aibotek Biotechnology Co., Ltd., Wuhan, China. The template cDNA was diluted in a 5-fold concentration gradient, and qRT-PCR was performed using the Real Universal Color PreMix (SYBR Green) Fluorescent Quantification Kit from Tiangen Biochemical Technology (Beijing) Co., Ltd. The qRT-PCR amplification conditions were as follows: initial denaturation at 95 °C for 3 min, followed by 40 cycles of 95 °C for 10 s, 60 °C for 30 s, and 72 °C for 30 s. Melting curve analysis was performed from 65 °C to 95 °C (0.5 °C increments, 5 s per step) after each run to confirm amplification specificity. Primer efficiency was determined using a standard curve generated from 5-fold serial dilutions of cDNA and ranged from 90% to 110%. The qRT-PCR reaction system and the reaction protocol were set according to the instructions.

For qRT-PCR analysis, three technical replicates were performed for each biological replicate. The qRT-PCR primers were synthesized by Fuzhou Shangya Biotechnology Co., Ltd. The genes involved in the quantitative real-time PCR used in this experiment included *Actin*, ethylene synthesis-related genes *FaACS* [50], *FaACO1*, and *FaACO2* [34]; ethylene signal transduction-related genes *FaETR1*, *FaETR2*, *FaETR13*, *FaEIN2*, and *FaEIN7* [35]; *FaERS1* [34], *FaERF13*, *FaERF118*, and *FaERF316* [51]. The primer sequences of these genes are shown in Table 1.

### 4.11. Data Processing

qRT-PCR results were normalized to the relative expression level of petals from ‘140’ strawberries at the large bud stage. The relative expression levels of genes were calculated using the relative quantification 2^−ΔΔCt^ method [52]. All statistical analyses were based on three biological replicates, with data presented as mean ± standard deviation (SD). ImageJ 2 was used to calculate the surface area, cell length, and cell width of petal cells. WPS 2019 was used for statistical data analysis. Origin 2024 and GraphPad Prism 9.5.0 were used for graphing. Adobe Photoshop 2023 was used to format the graphs.

## 5. Conclusions

This study provides a comprehensive analysis of petal senescence in the red-flowered strawberry cultivar ‘140’ across five developmental stages. Our findings demonstrate that cellular-level damage, particularly the progressive increase in epidermal cell breakage, precedes visible morphological wilting, with lower epidermal cells exhibiting higher susceptibility. The senescence process is driven by a synergistic interplay of continuous water loss, membrane lipid peroxidation, ROS accumulation, and the eventual imbalance of the antioxidant defense system, in which SOD and POD play key protective roles at late stages. At the molecular level, the ethylene signaling pathway appears to play a key regulatory role in petal senescence, as evidenced by the significant upregulation of *FaACO1* and multiple signaling genes (*FaETR1*, *FaETR2*, *FaEIN2*, *FaEIN7*, *FaERF13*, and *FaERF118)*, with *FaERF118* showing the highest and continuously increasing expression throughout senescence. These findings provide a theoretical foundation for extending flower longevity in ornamental strawberries. However, we acknowledge that the present study is correlative in nature and does not establish a causal role for ethylene or ABA; the apparent prominence of ethylene over ABA in terms of accumulation does not necessarily reflect its greater biological importance. Future research should employ functional validation through exogenous hormone treatments, inhibitor assays, or gene functional analyses, as well as transcriptomic approaches and genetic manipulation. These efforts will help elucidate the regulatory networks underlying petal senescence and develop practical strategies for enhancing ornamental value.

## Figures and Tables

**Table 1:** qRT-PCR primers used in this experiment.

Gene Name	Forward Primer (5′–3′)	Reverse Primer (5′–3′)
*Actin*	TGGGTTTGCTGGAGATGATG	CAGTTAGGAGAACTGGGTGC
*FaACS*	AACGAGTTTGGTTGGGATAA	GCAGGAACGATAGCGAAG
*FaACO1*	TACCTCAAGCACCTTCCTCGC	TTAGTGCCAAAGGTAGGACTA
*FaACO2*	GAAAGCACCTTCTTCTTGCG	CACCTTGGTACCAAAATTTGGT
*FaETR1*	GGTGACCTCATTCCCGTCTTT	ACAGGCCTCCATCAGAATTGA
*FaETR2*	CAAGGGATGCACTTCTACT	TGGCTGTTACTGACCCATT
*FaETR13*	CGGTGGTAGGAGGAACAACA	CGGTGCTACCACCTAGTTCA
*FaERS1*	CTTCAAGAGATTGGCGACCAC	GGATCCATTTCTGGGCTGAG
*FaEIN2*	GGTCTTCTGAGTTTGGGGCA	GAAGGTGGCACTAGCTTGGT
*FaEIN7*	GGGTGACCTTGGAGAGGTTG	ATCAATCCACAAGCCGCCAT
*FaERF13*	GGCGTATGACCGAGCTGCTT	TACCGGCTCAACGTTGTTGGA
*FaERF118*	GAAGAGGGTGGTGAGGATCA	GGACTGTTGGGGAAGTTGAA

## Data Availability

The data presented in this study are available in the article.
